# Irradiation of human oral mucosa by 233 nm far UV-C LEDs for the safe inactivation of nosocomial pathogens

**DOI:** 10.1038/s41598-023-49745-3

**Published:** 2023-12-16

**Authors:** Johannes Schleusener, Silke B. Lohan, Loris Busch, Daniela F. Zamudio Díaz, Nevin Opitz, Claudia Sicher, Tom Lichtenthäler, Kerstin Danker, Steffen Dommerich, Thomas Filler, Martina C. Meinke, Paula Zwicker

**Affiliations:** 1 Center of Experimental and Applied Cutaneous Physiology, Department of Dermatology, Venereology and Allergology, Charité – Universitätsmedizin Berlin, Corporate Member of Freie Universität Berlin and Humboldt-Universität zu Berlin, Charitéplatz 1, 10117 Berlin, Germany; 2 Department of Pharmaceutics and Biopharmaceutics, Philipps-Universität Marburg, Robert‑Koch‑Str. 4, 35032 Marburg, Germany; 3 Technische Universität Berlin, Institute of Food Technology and Food Chemistry, Gustav-Meyer-Allee 25, 13355 Berlin, Germany; 4 Institute of Hygiene and Environmental Medicine, University Medicine Greifswald, Ferdinand‑Sauerbruch‑Str., 17475 Greifswald, Germany; 5 Institute of Biochemistry, Charité – Universitätsmedizin Berlin, Corporate Member of Freie Universität Berlin and Humboldt Universität zu Berlin, Charitéplatz 1, 10117 Berlin, Germany; 6 Department of Otorhinolaryngology, Charité – Universitätsmedizin Berlin, Corporate Member of Freie Universität Berlin and Humboldt-Universität zu Berlin, Charitéplatz 1, 10117 Berlin, Germany; 7 Ferdinand-Braun-Institut (FBH), Gustav‑Kirchhoff‑Str. 4, 12489 Berlin, Germany

**Keywords:** Infection, DNA

## Abstract

The inactivation of multi resistant pathogens is an important clinical need. One approach is UV-C irradiation, which was previously not possible in vivo due to cytotoxicity. Recently, far UV-C irradiation at λ < 240 nm was successfully used on skin with negligible damage. A potential application site is the nasal vestibule, where MRSA accumulates and cannot be treated using antiseptics. We irradiated 3D mucosa models and excised human mucosa with 222 and 233 nm far UV-C in comparison to 254 nm and broadband UV-B. Eradication efficiency was evaluated by counting colony forming units; irritation potential was evaluated by hen’s egg-chorioallantoic membrane assay and trans epithelial electrical resistance; cell viability was assessed by MTT. DNA damage and cell protective mechanisms were evaluated immunohistopathologically. On mucosa models, MRSA reduced by ≈ 5 log_10_ for 60 mJ/cm^2^ irradiation at 233 nm. A slightly increased cell viability was observed after 24 h. Lower doses showed lower irritation potential than the positive controls or commercial mouthwash, while 80 mJ/cm^2^ had strong irritation potential. DNA damage occurred only superficially and decreased after 24 h. On excised human mucosa, < 10% of keratinocytes were affected after 150 mJ/cm^2^ 222 nm or 60 mJ/cm^2^ 233 nm.

## Introduction

The use of UV-C irradiation has been shown to be effective for the inactivation of multi-resistant pathogens^1^, which is currently a major clinical concern^2^. The advantage is to avoid the formation of resistances^3^ compared to the use of classical disinfectants or antiseptics, such as Chlorhexidine and Triclosan or antibiotics, such as Mupirocin^4^, which reduces their efficacy^5–7^. In particular, the prophylactic use of topical antimicrobials, such as mupirocin, may be a cause of increasing bacterial resistance rates^8^. Therefore, alternative methods for Methicillin-resistant *Staphylococcus aureus* (MRSA) decolonization are needed.

There is currently no evidence that pathogen resistances or tolerance can be induced following to UV-C irradiation^9^. Other conceivable applications of a far UV-C treatment could be the direct inactivation of SARS-CoV-2^10^ in the mucous membrane of the throat or as an antiseptic treatment during surgery. Successful eradication of other viruses^11^, or fungi^12^ has also been demonstrated, recently.

Conventionally, UV-C sources at 254 nm are used for the disinfection of surfaces^13^, instruments^14^ or room air^15,16^ in shielded air cleaners that prevent human exposure. This is crucial, as UV-C radiation at 254 nm is highly cytotoxic, reduces cell viability^17^ and causes a large amount of DNA damage. The main DNA damage are cyclobutane pyrimidine dimers (CPD) and pyrimidine-pyrimidone photoproducts (6-4PPs). Due to the faster repair mechanisms in 6-4PPs^18,19^, CPDs in particular are linked to the development of cancer^20,21^.

Recently, several studies have shown the safe application of far UV-C eradication using λ < 240 nm to the skin^22–24^. The underlying concept is based on the strong absorption of UV-C light at λ < 245 nm by proteins and a consequently lower penetration depth^25^, entailing an almost complete attenuation in the *Stratum corneum*, which does not contain cell nuclei^26^ and therefore, does not show DNA damage. Due to the small size of microbes, the disinfection efficiency is only minimally reduced, as shown for MRSA on 3D murine skin in vivo^26^, airborne^11^ or on human skin^24^ in vivo.

More recently, also 233-nm-LED-sources were applied showing negligible skin damage^27^. The advantage of using far UV-C LEDs is operation at low voltages and the potential of manufacturing miniaturised light sources, which is not possible with 222 nm excimer lamps. Regarding the slightly lower absorption and hence deeper penetration of 233 nm compared to 222 nm, different applications could be useful for both radiation sources. In this regard, 222 nm has some advantages for room air disinfection, resulting in chronic irradiation of healthy people, where minimal skin damage is acceptable. In contrast, 233 nm has some advantages in antiseptic applications, such as treatment of chronic wounds or intraoperative inactivation of pathogens, where a small amount of damage can be justified for successful treatment of patients.

Manufacturing miniaturised far UV-C light sources enables the endoscopic application in hollow body cavities, thus expanding the range of potential medical treatments. LED-light sources have already been used in the UV-B range to treat oral bacteria^28^, although the level of DNA damage has not been analysed.

The primary location of MRSA and Methicillin-sensitive *Staphylococcus aureus* (MSSA) is the nasal vestibule, which can be considered an initial point for colonization of the body^29^. This location is virtually inaccessible using topical antimicrobial treatment. One successful approach in eradicating multi resistant pathogens has been antimicrobial photodynamic therapy for bacterial decolonisation using a methylene-blue based photosensitizer and irradiating the anterior nares with 9 J/cm^2^ red light (665 nm) to generate reactive oxygen species^30^. A variation of the device might also reach deeper areas of the throat. However, as the technique depends on the topical application of a photosensitizer, it may not be practical in these locations.

Therefore, this location is especially interesting for the application of far UV-C irradiation. Bactericidal efficiency on human oral bacteria has previously only been evaluated using broadband and narrowband UV-B light^28^.

In comparison to promising results using this technology on reconstructed human epidermis models and excised human skin ex vivo^27^, human oral mucosa from the nasal vestibule does not contain a stratum corneum. Nevertheless, mucin, secreted by mucosa, shows strong absorption in the far UV-C range and therefore provides a protection to DNA damage in mucosa induced by far UV-C radiation^27^.

The aim of this study was to evaluate the inactivation efficiency and side effects on mucosa by the far UV-C irradiation sources 233 nm in comparison to 222 nm, 254 nm (near UV-C) and broadband UV-B irradiation in order to assess the potential for applications in the nasal vestibule. This evaluation was carried out by determining the following outcome measures:

- The eradication efficiency of microbes was evaluated by suspending bacteria on 3D mucosa models and counting CFUs on incubated tryptic soy agar (TSA) plates after 24 h.
- The irritation potential was evaluated by using a hen's egg-chorioallantoic membrane (HET-CAM) assay on pathogen free eggs and trans epithelial electrical resistance (TEER) on the cultured 3D mucosa models.
- The cell viability was determined using an MTT assay on 3D mucosa models.

The DNA damage and cell protective mechanisms were evaluated on three different models using immunohistopathology:

- 3D mucosa models (Model A) were used to determine instantaneous effects.
- Repair mechanisms were analysed on a limited number of full-thickness mucosa equivalents of immortalised cells (Model B) using only 233 nm, as these experiments required longer cultivation times than at the time achievable with model A.
- Reconstructed mucosa models show a high homogeneity and viability, but to achieve a higher morphological similarity to human mucosa in vivo, excised human oral mucosa was additionally used.

## Results and discussion

### Eradication of microbes (Model A)

**Figure 1:**
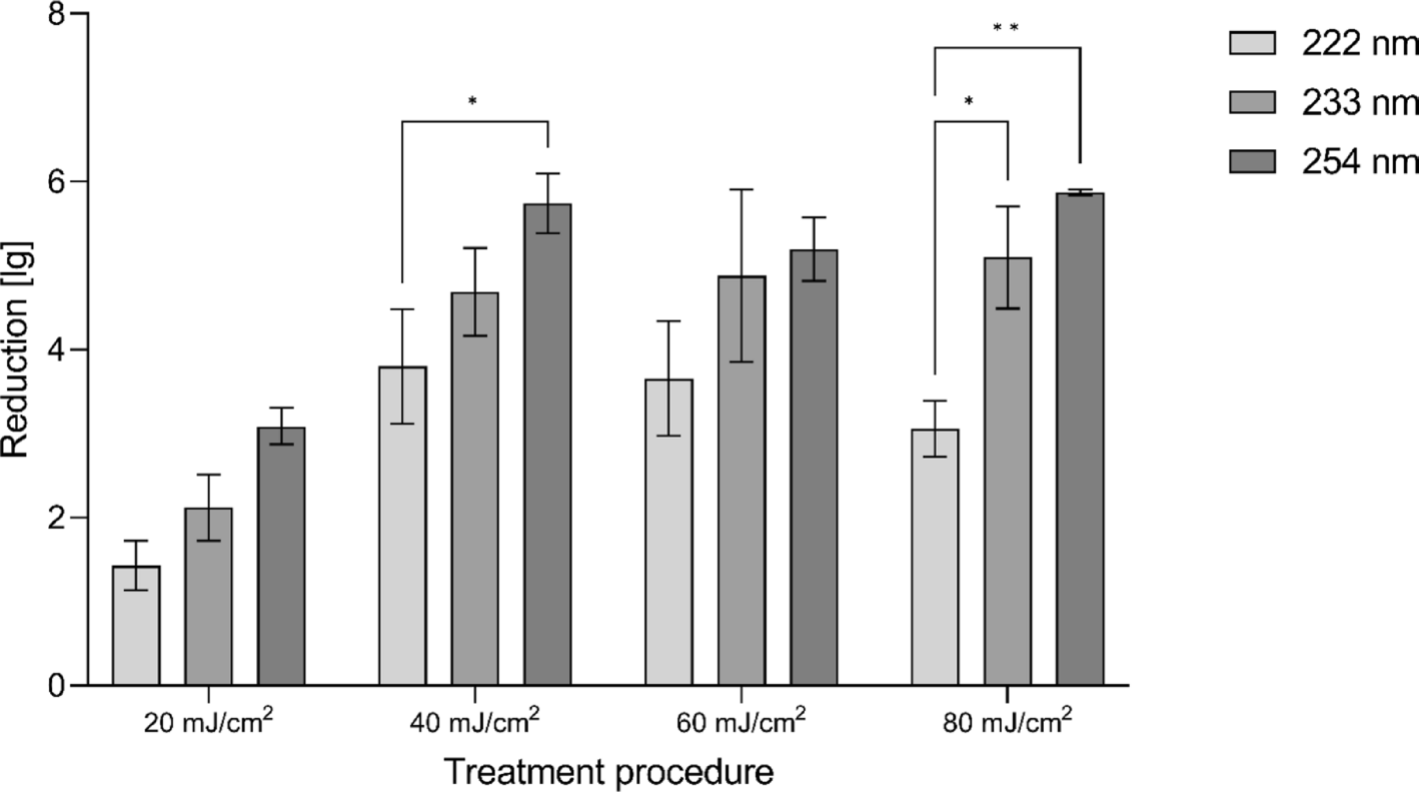
Microbial reduction of MRSA on mucosa models (Model A). Log_10_ reduction factor of microbes on 3D mucosa models (Model A) irradiated at 222, 233, and 254 nm using doses of 20, 40, 60 and 80 mJ/cm^2^ (Mean ± SEM, *n* = 3–5. *p < 0.05, **p < 0.01).

The eradication of microbes was performed on 3D mucosa models. The reduction factors are shown in Fig. 1 for irradiation with 222, 233 and 254 nm with doses of 20, 40, 60 and 80 mJ/cm^2^. The recovery rate of bacteria was about 89 ± 21%.

It can be observed that the reduction factor increased with wavelength for almost all doses; only for 60 mJ/cm^2^ it is almost identical at 233 and 254 nm. As the dose increased from 20 to 40 mJ/cm^2^, the reduction factor increased for all applied wavelengths, but a further increase to 60 and 80 mJ/cm^2^ showed no considerable differences for 233 and 254 nm as the applied microorganisms were almost completely reduced. Although the reduction of bacteria on the mucosa models was lower than on carriers as previously shown before^27^, an in vivo application is still promising. Lower reduction may result from an uneven surface mimicking natural conditions, as well as from a reduced amount of radiation reaching the surface due to the insert surrounding the mucosa model; under realistic conditions, the skin area to be treated was not shielded by plastic, allowing the radiation to reach the bacteria. Furthermore, a reduction of about 5 log_10_ levels was reached using the mucosa models when applying 60 mJ/cm^2^ (233 nm). The irradiation was therefore sufficient to eradicate the bacteria.

The study has some important limitations. Bacteria colonizing the nose grow in biofilms. However, little is known about biofilm thickness or the number of bacteria in a defined area. That is why a high number of planktonic cells was used in the presented test setup. Since the interest of the study was to investigate the impact of the surface structure on the bacteria inactivation, bacteria colonized the surface but biofilm formation was not considered.

### Compatibility and irritation potential (Model A)

**Figure 2:**
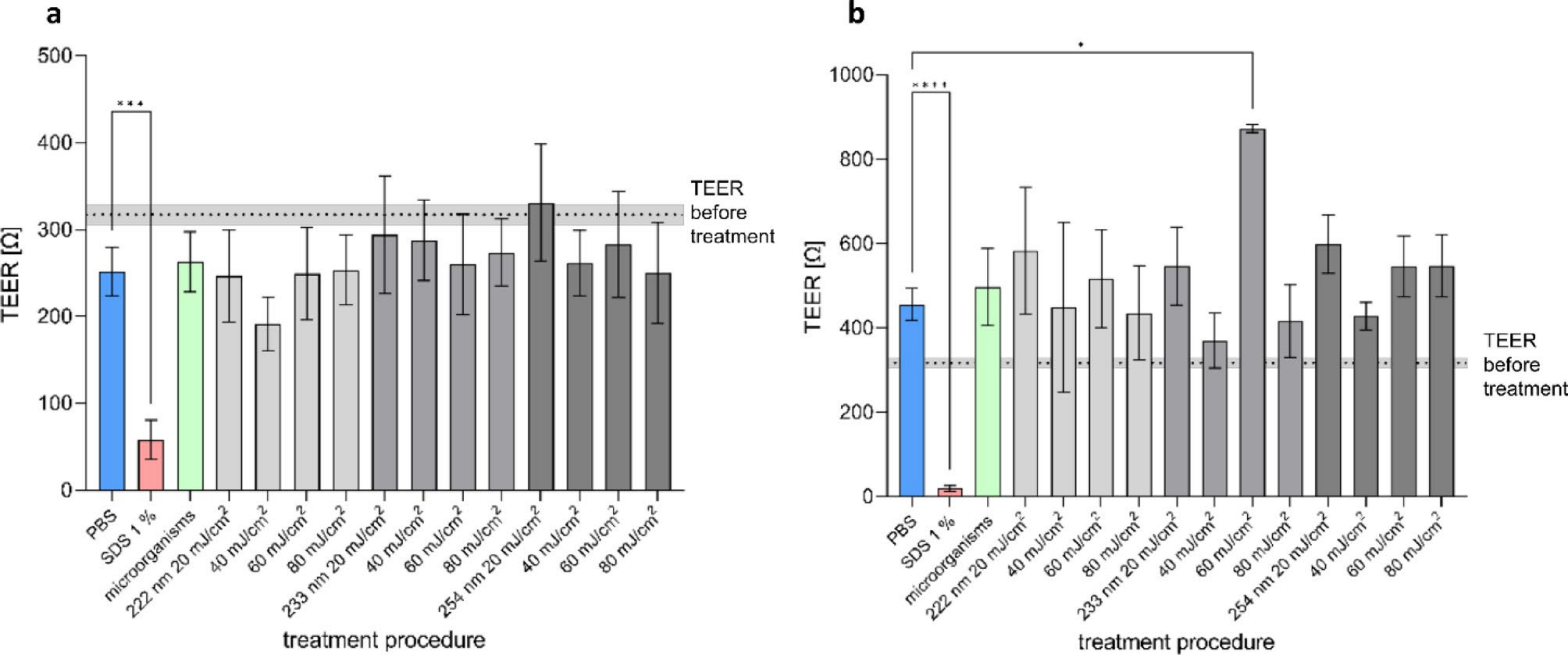
Trans epithelial electrical resistance of mucosa models (Model A). TEER [Ω] directly (**a**) and 24 h (**b**) after irradiation of the mucosa models (Model A) (Mean ± SEM, *n* = 2–17, control: *n* = 133. *p < 0.05, ***p < 0.001, ****p < 0.0001).

Directly after irradiation, the TEER was not significantly affected (Fig. 2). Furthermore, also 24 h after irradiation, no differences to the non-irradiated control were observed. The irritation potential was determined by using the HET-CAM assay evaluating strength of reactions according to the ICCVAM test protocol^31^.

**Figure 3:**
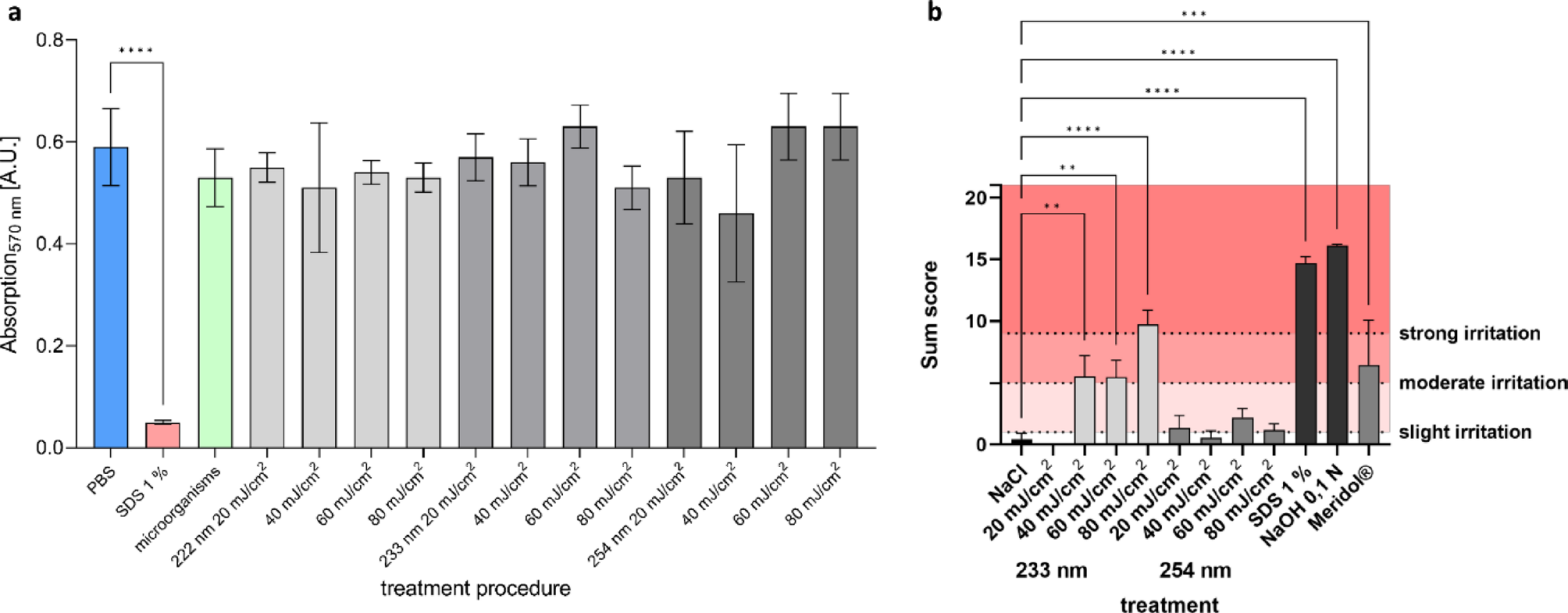
Cell viability and irritation of mucosa models (Model A). (**a**) Absorption of purple coloured formazan produced by viable cells within the MTT assay 24 h after irradiation with 222, 233 and 254 nm and doses of 20–80 mJ/cm^2^ (Mean ± SEM, *n* = 2–7). (**b**) Sum score reached by various treatments of the CAM by 233 and 254 nm irradiation, as well as treatment with 1% SDS, NaOH 0.1 N and Meridol^®^ mouthwash. Sum score is composed of single scores for haemorrhage, vessel lysis and coagulation (Mean ± SEM, *n* = 3–4 with 2–3 technical replicates each; **p < 0.01, ***p < 0.001, ****p < 0.0001).

During the experiments for reduction of microbes, performed on 3D mucosa models, the cell viability was not affected within 24 h after irradiation (Fig. 3a), independently of the treatment with PBS or the bacteria suspension. Since the mucosa models were washed several times after treatment to recover the bacteria, no microbial contamination had impact on cell viability and data could be combined.

Irradiation at 254 nm (20–80 mJ/cm^2^) and 233 nm (20 mJ/cm^2^) showed no or only slight irritation potential, while irradiation at 233 nm (40–60 mJ/cm^2^) revealed moderate irritation potential (Fig. 3b). Irradiation at 233 nm (80 mJ/cm^2^) had a strong irritation potential, which was not as strong as for the positive controls. One reason for this could be the increased absorption of radiation by proteins, leading to a direct effect when using 233 nm UV-C radiation. In contrast, 254 nm radiation is mainly absorbed by DNA/RNA leading to a long-lasting effect, not directly visible after treatment. Treatment with a commercially available mouthwash (Meridol^®^), which is recommended for daily use, caused irritation comparable to 40–60 mJ/cm^2^ of 233 nm irradiation.

### DNA damage

#### 3D mucosa models (Model A)

**Figure 4:**
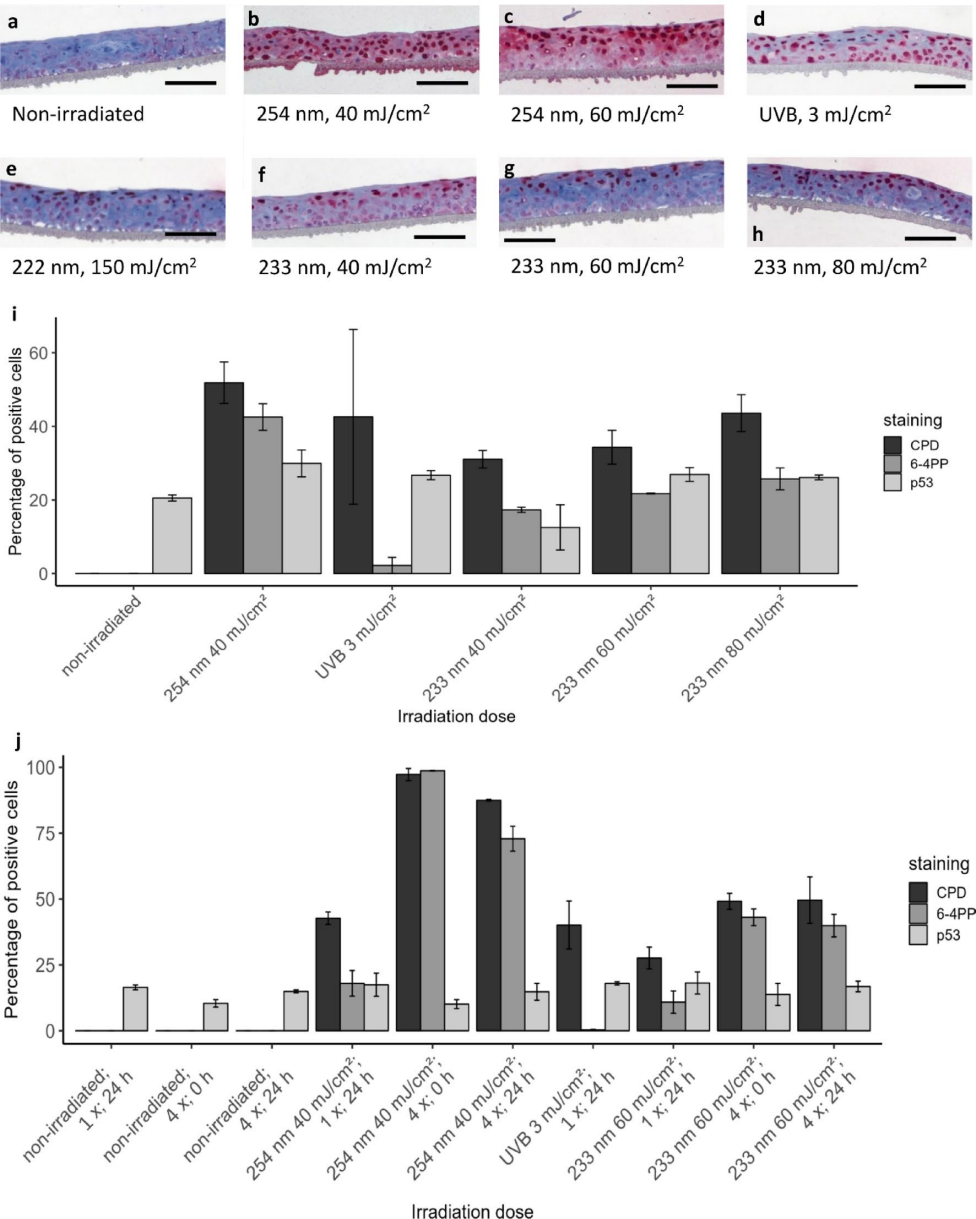
DNA damage in mucosa models (Model A). Immunohistological sections of 3D mucosa models stained for CPD damage. Non-irradiated (**a**), irradiated at 254 nm–40 mJ/cm^2^ (**b**), 254 nm–60 mJ/cm^2^ (**c**), broadband UV-B–3 mJ/cm^2^ (**d**), 222 nm–150 mJ/cm^2^ (**e**), 233 nm–40 mJ/cm^2^ (**f**), 233 nm–60 mJ/cm^2^ (**g**) and 233 nm–80 mJ/cm^2^ (**h**), Scale bar: 100 µm. DNA damage of 3D mucosa models (Model A) fixated directly after (**i**), as well as directly (0 h) and 24 h after single (1 ×) and multiple (4 ×) irradiation (**j**). CPD, 6-4PP and p53 (Mean ± SEM, 1 × 24 h: *n* = 3, 4 × 0 h: *n* = 3, 4 × 24 h: *n* = 2–3).

The 3D mucosa models were analysed immunohistopathologically for DNA damage and induction of cell protective mechanisms. Figure 4 shows sections of the irradiated samples and a non-irradiated control, where CPD damage is indicated by the dark-red stained cells. While CPD damage occurred throughout the epithelium in 3D models irradiated with 254 nm and UV-B, only superficial cells were affected by far UV-C irradiation at 222 and 233 nm.

The percentage of cells in the model, that showed DNA damage (CPD and 6-4PP), and induction of cell protective mechanisms (p53) directly after irradiation is shown in Fig. 4i. The amount of CPD damage after 233 nm irradiation increased with increasing dose and at 80 mJ/cm^2^ reached the level of 3 mJ/cm^2^ UV-B radiation (> 40%), but remained less than 40 mJ/cm^2^ at 254 nm. Significant differences were not observed. This seemingly differed from the results of a previous study on reconstructed human epidermis models using identical instrumentation and doses^27^, where irradiation at 222 and 233 nm only showed a negligible amount of CPD damage, whilst the damage at 254 nm was > 40% and at UV-B irradiation > 90%.

There was also an increase in 6-4PP damage with increasing dose after 233 nm irradiation, but it did not reach the level of 254 nm irradiation (> 42%). With 3.8%, the amount of 6-4PP damage at 3 mJ/cm^2^ UV-B irradiation was considerably low.

The p53 suppressor gene can be used as a marker for the loss of the protective function of keratinocytes and melanocytes against genotoxic damage. However, this is a rather long-term effect and is unlikely to occur immediately after irradiation. Therefore, inactivation and mutation of p53 in keratinocytes following UV exposure has already been demonstrated^32^, but could not be verified directly after irradiation, here. As a short-term effect, p53 is rather a marker for protection and would be expected to increase subsequent to irradiation, which was observed for 233 nm with doses ≥ 60 mJ/cm^2^. Increase of p53 can primarily indicate cell cycle arrest related to DNA repair. A further accumulation could indirectly also indicate apoptosis.

Since UV induced DNA damage can be rapidly reversed by the enzymatic repair system^33^ or apoptosis^34^, some of the irradiated samples were incubated in culture medium at 37 °C for 24 h in culture medium after irradiation. In addition, possible accumulating effects were analysed by multiple irradiation (4 × every 24 h) at the same dose (Fig. 4j).

An accumulating effect of multiple irradiation for CPD damage was observed for 254 nm (40 mJ/cm^2^) and 233 nm (60 mJ/cm^2^), while for the latter irradiation, the increase after multiple irradiation was only small. A similar behaviour was observed for 6-4PP damage.

The decrease of CPD damage, when incubating further 24 h after irradiation was only low, compared to previous results on reconstructed human epidermis models^27^. As the 3D mucosa models were cultured from tumour cells (TR146), the repair mechanisms could possibly be reduced compared to human mucosa.

Efficient removal after a few days and complete repair after several weeks of DNA damage in excised mucosa of human subjects irradiated with UV-B has been observed^35^. For human skin, repair of DNA damage on the time scale of 1–10 days has been reported^22,36,37^.

The nucleotide excision repair pathway has been identified as a primary repair mechanism of DNA damage in mucosa^38^, which was observed to be lower in oral tissue compared to skin, and, therefore, could lead to the accumulation of DNA damage^39^. In a later study on 3D models, irradiated with UV-B, DNA repair and the amount of apoptotic cells were significantly lower in gingival and oral tissues compared to human skin^40^, which is in agreement with our findings.

Only after irradiating 4 × at 40 mJ/cm^2^ of 254 nm and fixating immediately afterwards, an obvious decrease of p53 was observed. Significant differences could not be determined.

#### Full-thickness mucosa equivalents of immortalised cells (Model B)

**Figure 5:**
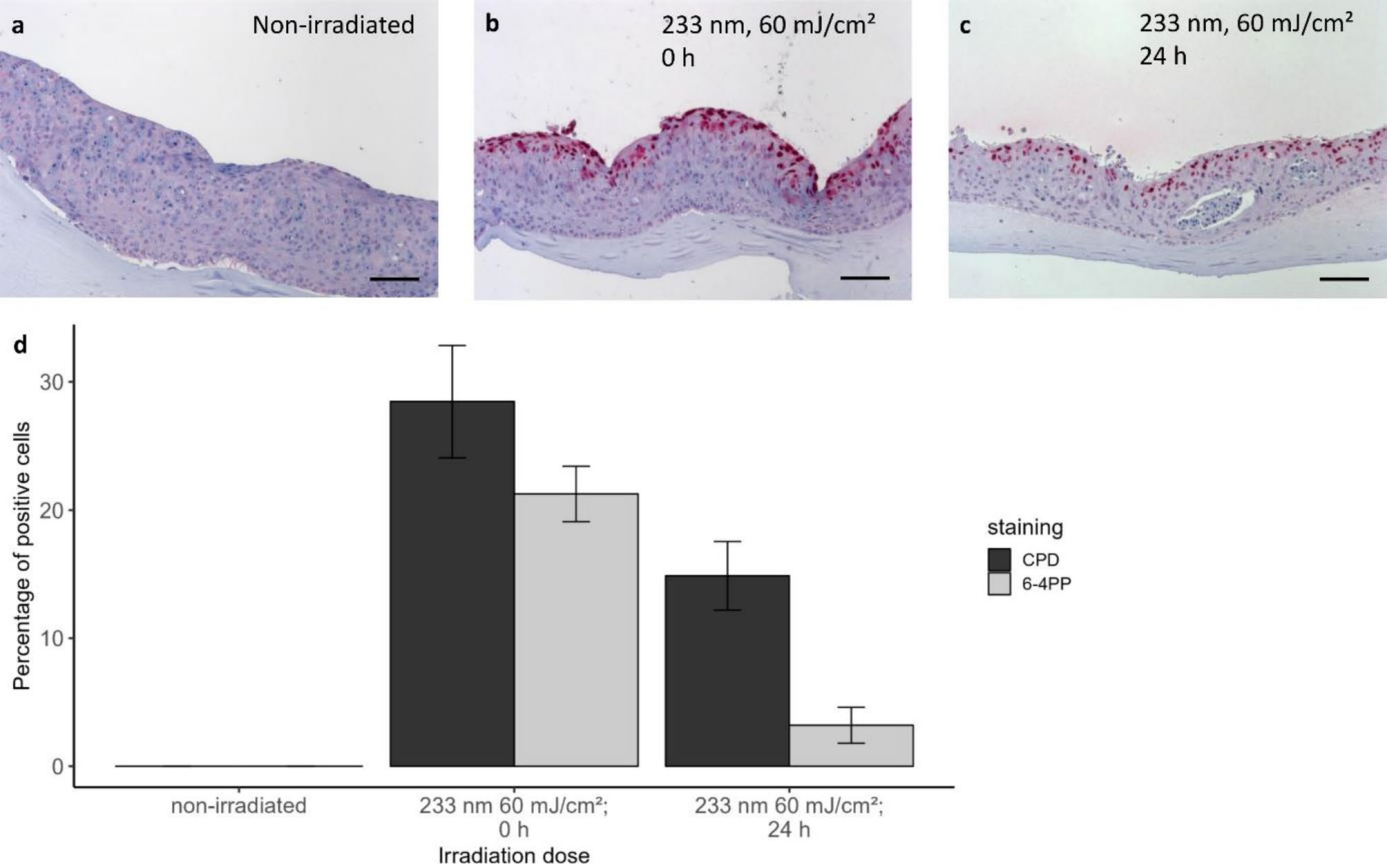
DNA damage in mucosa models (Model B). Immunohistological sections of full-thickness mucosa equivalents of immortalised human gingival keratinocytes and fibroblasts (Model B) stained for CPD damage, non-irradiated (**a**), fixated directly (**b**, 0 h) and 24 h after single irradiation (**c**) Scale bar: 100 µm. (**d**) DNA damage on full-thickness mucosa equivalents of immortalised human gingival keratinocytes and fibroblasts fixated directly (0 h) and 24 h after single irradiation. CPD and 6-4PP (Mean ± SEM, *n* = 3).

The reduced repair mechanisms, when fixating 24 h after irradiation, could be due to decreased cell viability. The experiments for DNA damage were performed after the models had been transported from Greifswald to Berlin, so they may have suffered along the way. Therefore, additional irradiation experiments for DNA damage were performed on mucosa models (*n* = 3) cultured from non-transformed immortalised keratinocytes and fibroblasts and were fixated immediately (Fig. 5a–c), and 24 h after irradiation.

Figure 5d shows a slightly lower DNA damage immediately after exposure to 60 mJ/cm^2^ at 233 nm of the mucosa models from immortalised cells (model B), compared to model A. This could also be due to a thicker epithelium of model B compared to model A. After 24 h, the level of CPD damage of model B nearly halved, while the amount of 6-4PP damage even decreased by a factor of > 6. These results demonstrate the potential of repair mechanisms for model B following far UV-C irradiation. Due to the presence of fibroblasts, model B is closer to the in vivo condition of human oral mucosa.

#### Excised human oral mucosa

**Figure 6:**
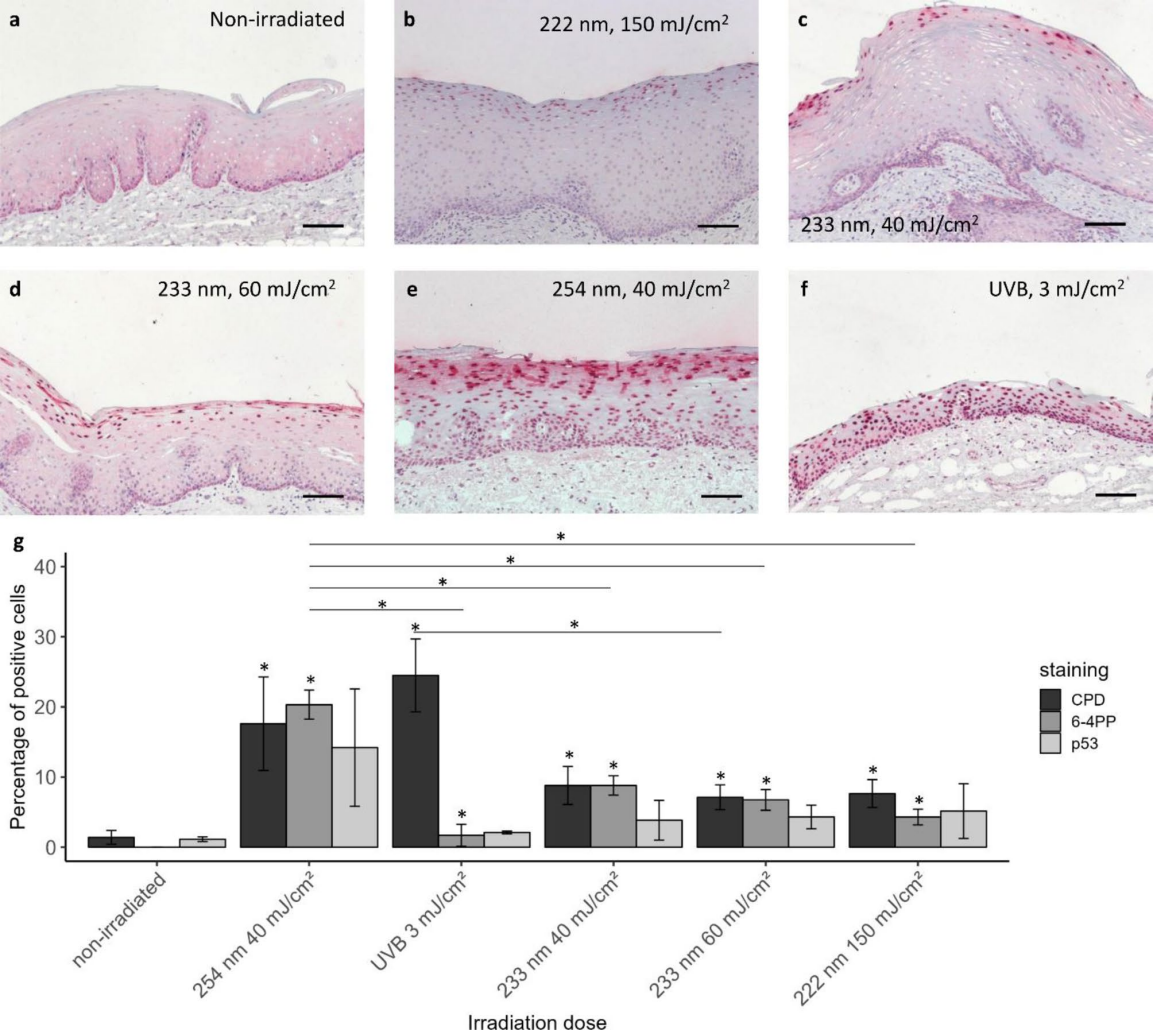
DNA damage in excised human oral mucosa. Immunohistological sections of excised human oral mucosa stained for CPD damage. Non-irradiated (**a**), irradiated at 222 nm–150 mJ/cm^2^ (**b**), 233 nm–40 mJ/cm^2^ (**c**), 233 nm–60 mJ/cm^2^ (**d**), 254 nm–40 mJ/cm^2^ (**e**) and broadband UV-B–3 mJ/cm^2^ (**f**) Scale bar: 100 µm. DNA damage of excised human oral mucosa (**g**). CPD, 6-4PP and p53 (Mean ± SEM, *n* ≥ 4, *p ≤ 0.05 between groups or compared to non-irradiated (above bars)).

As the behaviour of the 3D mucosa models might be limited regarding CPD damage, excised human mucosa has been obtained and was irradiated ex vivo. Immunohistological images of excised human mucosa stained for CPD damage are shown in Fig. 6a–f. Similar to the 3D mucosa models, the CPD damage for 40 mJ/cm^2^ 254 nm and 3 mJ/cm^2^ UV-B reached considerably deeper compared to 222 and 233 nm, where only a superficial part of the epithelium was affected. However, as the epithelium of excised human mucosa is more than twice as thick as that of 3D mucosa models, the percentage of the CPD damage for excised human mucosa (Fig. 6g) was considerably lower compared to those for 3D mucosa models.

The CPD damage (Fig. 6g) was < 25% after 3 mJ/cm^2^ UV-B-, < 20% after 40 mJ/cm^2^ 254 nm- and < 10% after far UV-C irradiation of 222 nm (150 mJ/cm^2^) or 233 nm (40 and 60 mJ/cm^2^). This trend is comparable to the findings previously observed for excised human skin using the identical instrumentation^27^. An increase of DNA damage with increasing dose from 40 to 60 mJ/cm^2^ after 233 nm irradiation was not observed.

The increase of CPD and 6-4PP damage compared to non-irradiated mucosa was significant for all doses, changes in p53 were not significant (Fig. 6g).

The lining mucosa, which makes up most of the oral mucosa, does not contain a protective stratum corneum, as skin does. However, strong absorption in the far UV-C range of the mucin-containing saliva, covering the mucosa, has been shown^41^, which could induce a protective effect.

In a recent study using identical irradiation on human skin, the authors claimed, that DNA damage due to 3 mJ/cm^2^ UV-B irradiation, which is ≈ 10% of a minimal erythema dose (MED) for UV-B for skin type II (Fitzpatrick), is likely not avoidable, due to daily sunlight exposure.

This claim cannot be maintained for large parts of the nasal vestibule and a low amount (≪ 1 MED) of UV-B irradiation for vitamin D generation^42^ can therefore not be considered necessary. Further, a threshold of harmless UV exposure does not exist^36,37^. However, the DNA damage observed in excised human mucosa is low enough to justify medical applications, e.g. the eradication of nosocomial pathogens.

## Conclusion

The results indicate, that far UV-C irradiation at 222 and 233 nm induces only a low level of mucosal irritation, which is acceptable for a clinical use. Therefore, a potential application to eradicate nosocomial pathogens seems promising. While the doses applied at 222 nm cause slightly less DNA damage compared to 233 nm, the antimicrobial efficiency is higher at 233 nm.

## Methods

### Cell culture

#### Model A

Cells derived from human squamous cell carcinoma, TR146, were cultured in DMEM/F12 cell culture medium with addition of 2 mM Glutamine (both PAN-Biotech GmbH, Aidenbach, Germany) and 10% fetal bovine serum (FBS, Gibco Thermo Fisher Scientific, Waltham, MA) at 37 °C in a humidified atmosphere (5% CO_2_).

When a confluence of 80% was reached, cells were detached with 0.05% trypsin/0.02 mM EDTA after rinsing with PBS without Ca^2+^/Mg^2+^ (woPBS, ccPro, Vogtei, Germany). Morphology was checked regularly by microscopy. The cells were free of mycoplasma. Viability of cells was ensured by trypan blue exclusion.

#### Model B

For additional experiments, hTERT-immortalised human gingival keratinocytes (OKG4/bmi1/TERT) kindly provided by Susan Gibbs^43–45^ and immortalised gingival fibroblasts (IhGF) were used to prepare full-thickness mucosal equivalents. OKG4 cells were cultured on collagen IV coated cell culture flasks with serum-free growth medium DermaLife K containing 1% penicillin–streptomycin (P/S). Medium was changed every two days and cells were passaged at 60–70% confluence. IhGF were cultured in DMEM containing 10% FCS with 1% glutamine and 1% P/S and passaged at a confluence of 70–80%.

### 3D mucosa models

#### Model A

Preparation of 3D mucosa models was conducted as already published^46^. In short, after trypsinisation, cells were resuspended in Medium A composed of 485 ml DermaLife^®^ basal medium (CellSystems GmbH, Troisdorf, Germany) with addition of 5 ml human keratinocyte growth supplement (Life Technologies, Carlsbad, CA), 0.01 mg/ml Gentamicin (GE Healthcare, Chicago, IL) and 0.25 µg/ml Amphotericin B (PAN-Biotech GmbH, Aidenbach, Germany) until a cell density of 1 × 10^6^ cells/ml was reached. In a 6-well cell culture plate, 500 µl of Medium A were dropped in the middle of each well and cell culture inserts (MilliCell 0.4 mm PCF, 12 mm diameter, Merck Millipore, Burlington, MA) were placed onto this droplet. Into the insert, 300 µl of the cell suspension were added. After 24 h incubation, 1 ml Medium A was added to the well and the medium in the inserts was renewed. After further incubation for 24 h, the medium was renewed in the inserts and the well. The next day, cells were exposed to the air for drying for 10 min and afterwards, Medium B (Medium A + 0.09 mM CaCl_2_) was provided to the cells only from basolateral (airlift culture day 0). The medium was renewed every other day for further 14 days.

For measurement of the trans epithelial electrical resistance (TEER), inserts were placed in 24-well plates with 600 µl PBS (with Ca^2+^/Mg^2+^) and washed with 400 µl PBS. Fresh PBS was added, and TEER was measured with the EVOM™ Epithelial Voltohmmeter with a 5TX2 electrode (World Precision Instruments Ltd.).

Cell viability was analysed using Thiazolyl Blue Tetrazolium Bromide (MTT, Sigma Aldrich, St. Louis, Missouri, USA). Every insert was placed in 500 µl cell culture medium with MTT (0.5 mg/ml) and incubated for 3 h. Afterwards, purple coloured formazan was solubilised by transferring the inserts to 2 ml elution solution (2-Propanol, 0.04 M HCl). After darkened overnight incubation at 4 °C, 200 µl of the supernatant were transferred to a 96-well plate and absorption was measured at 560 nm.

For immunohistochemical analysis of DNA damage, inserts were placed on 1.4 ml ultrapure agarose (1% in water/Medium B, Invitrogen, Waltham, MA) on day 9 of airlift culture and subsequently sent from Greifswald to the Charité, where they were further cultivated in 6-well plates at 37 °C, 5% CO_2_ and 100% humidity in OS Rep-Air–liquid interface medium (OS-Rep-ALI, CM-125, Henkel AG & Co. KGaA, Düsseldorf, Germany) until day 14. The medium was changed every 2 days. Due to photosensitivity of the medium, the mucosa models were placed on PBS during irradiation.

#### Model B

For additional experiments, the full-thickness oral mucosa equivalents were generated as previously described^47^. Briefly, 1.2 ml type I bovine collagen solution (Advanced BioMatrix, San Diego, CA, USA) was mixed with 300 µl 10 × HBSS (Gibco™, Waltham, MA, USA), and 100 µl 2N sodium hydroxide (AppliChem, Darmstadt, Germany). 300 µl FCS (Biochrom, Berlin, Germany) containing 3 × 10^5^/ml fibroblasts and 1.125 ml sterile H_2_O were then added to this solution. For polymerisation, 2.5 ml of the fibroblast-collagen I-mixture was transferred to a culture plate insert (24 mm diameter, pore size: 0.4 µm; Corning Costar^®^, Corning, NY, USA) placed in 6-well-plates (Corning, Corning, NY, USA), and incubated in a humid box at 37 °C for 2 h. Then, the gel was cultivated in DMEM containing 10% FCS. The next day, the medium was removed and 4.2 × 10^6^ OKG4 cells in DermaLife K medium containing 60 µM Ca^2+^ were placed on the gel. After reaching confluence, the mucosal equivalent was subjected to an air-lift by withdrawing medium from the top and replacing the medium in the well with DermaLife K medium containing 1.4 mM Ca^2+^. The medium was changed every other day. On day 5 after air-lift, the equivalents were irradiated.

### Antimicrobial efficacy testing and UV-C irradiation

A bacterial suspension with *Staphylococcus aureus* DSM 11822 (MRSA) was prepared by detaching bacteria from tryptic soy agar (TSA)-plates (Carl Roth, Karlsruhe, Germany) after 24 h incubation at 37 °C by use of 2 ml PBS. Bacteria were washed three times with PBS following centrifugation at 10,000×*g* for 1 min and final centrifugation at 7150×*g* for 3 min. The suspension was adjusted to an OD_620_ of 0.1–0.15 being equivalent to 1–5 × 10^8^ colony-forming units (cfu)/ml and diluted 10 × with PBS.

The mucosa models were treated with 50 µl of a bacteria suspension (1–5 × 10^7^ cfu/ml), which is a relatively high cell count that represents a worst-case scenario, considering an in vivo study that classified human volunteers with two consecutive nasal cell counts one week apart of > 10^3^ cfu to be persistent carriers^48^. Bacteria were allowed to settle down for 10 min in aseptic conditions at room temperature. In parallel, inserts without bacteria treatment but PBS addition were handled in the same way.

Afterwards, mucosa models were irradiated with doses of 20–80 mJ/cm^2^. Next, bacteria were removed by washing the inserts four times with 500 µl PBS. The PBS solution containing the bacteria was pooled and plated onto TSA-plates. Plates were incubated at 37 °C for 24 h and cfu were counted to calculate the log_10_ reduction of viable bacteria according to Eq. (1) where *n*_*c*_ is the number of cfu on control mucosa models without irradiation and *n*_*p*_ is the number of cfu on irradiated mucosa models.

1 $${log}_{10}\, reduction={log}_{10}\, {(n}_{c})-{log}_{10}\,({n}_{p}).$$

### Irritation potential

The hen’s egg test (HET) was used to test irradiation with 233 and 254 nm for its irritation potential. Specified pathogen free eggs (SPF, VALO BioMedia GmbH, Osterholz-Scharmbeck, Germany) were incubated for 2–10 days at 17 ± 3 °C upright after delivery. Afterwards, eggs were incubated at 37.5 ± 1 °C with a humidity of 62.5 ± 7.5% for 9 days. For 1 h per day, heating was stopped, and eggs allowed to cool down. During incubation, eggs were rotated 8 times per day for 2 h with 1 h rest in between.

On day 9, viability of the embryos was determined by candling and eggs were disinfected with ethanol, 70%. The air space was labelled. For the next 24 h, eggs were incubated upright with the blunt pole facing upwards without any further rotation.

On day 10, the egg shell was removed from the air space with a fine scissor and tweezers. The inner shell membrane was wetted with sodium chloride solution (0.9%, 500 µl NaCl) and removed.

Before treatment with NaCl (0.9%), positive controls (1% SDS, 0.1 N NaOH), or irradiation, a photo of the chorioallantoic membrane (CAM) was taken. The CAM was then treated with 300 µl test solution or 300 µl NaCl (0.9%) were added and irradiated accordingly. Five minutes after treatment (negative/positive control) or after irradiation (up to 9 min), the occurrence of three reactions was observed: lysis of vessels, intravascular or extravascular coagulation of blood and haemorrhage and the severity of these reactions.

The evaluation was conducted by microscopy using a Nikon SMZ 18 stereomicroscope with a P2-SHR Plan Apo 0.5 lens (NIS Elements D 5.30.03).

Each reaction and severity was assigned to one of the scoring categories first described by Luepke^49^ and recommended by the Interagency Coordinating Committee on the Validation of Alternative Methods (ICCVAM)^31,50^ (Table 1).

**Table 1:** Irritation scores assigned to factors of severity assessed by optical evaluation.

Effect	characteristic	Irritation score
Haemorrhage (bleeding of blood vessels)	Rarely	3
	Multiple	5
	Massive	7
Vessel lysis	Rarely	1
	Extensive	5
Intravascular coagulation	Rarely	5
	Multiple	7
	Massive	9
Extravascular coagulation	Increased opacity	9

For every egg, the irritation scores were summed up to build the sum score that was used for categorizing the irritation potential. A sum score of 0–0.9 indicates no irritation potential of a substance or treatment, whereas a sum score of 1–4.9 indicates slight, 5–8.9 moderate and 9–21 a strong irritation potential^31,49,50^.

### Human oral mucosa

Human oral masticatory and soft palate mucosa samples were obtained from the Clinic for Ear, Nose and Throat Medicine and the Institute of Pathology, both Charité – Universitätsmedizin Berlin to analyse DNA damage subsequent to irradiation and were jointly analysed. In total, samples from 10 different donors were obtained, but due to their small size, not all combinations of excitation source and dose could be studied on each sample. All experiments were performed within 3 h after surgery and were immersed in 0.9% NaCl in between. The irradiation experiments were performed in standardised laboratory conditions at 21 °C. 3 mm punch biopsies were placed on wet paper tissue to prevent drying out. The experiments were approved by the Ethics Committee of the Charité – Universitätsmedizin Berlin (EA1/324/19). Informed written consent was obtained from the donors, no samples were procured from prisoners and all procedures complied with the Declaration of Helsinki.

### UV irradiation

The irradiation experiments were performed using a 233 nm far UV-C light source (12 nm FWHM), consisting of 120 LEDs and a distributed Bragg reflector short pass filter suppressing wavelengths > 240 nm (Ferdinand-Braun-Institut, Berlin, Germany)^51,52^. A homogenous irradiance of 0.13 mW/cm^2^ was achieved on a 70 mm × 70 mm area.

Further, a 222 nm Kr-Cl Excimer lamp with a short pass filter suppressing wavelengths > 230 nm and an irradiance of 0.27 mW/cm^2^ was used (ExciJet222 30-130 Kit (111073), USHIO Deutschland GmbH, Steinhöring, Germany).

As positive controls, two light sources with emission wavelength > 245 nm were used: a 254 nm mercury gas discharge lamp with an irradiance of 0.52 mW/cm^2^ for analysis of DNA damage and 0.29 mW/cm^2^ for analysis of microbe reduction (LPL-R-01, sglux GmbH, Berlin, Germany) and a UV-B hand lamp with a 0.04 mW/cm^2^ irradiance in the 280–400 nm range, also containing a small amount of UV-A (TH-1E; Cosmedico Medizintechnik, Stuttgart, Germany).

For the UV-C light sources, the irradiance was measured using a UV radiometer with a SiC UV-C sensor (SXL55, sglux GmbH, Berlin, Germany). UV-B irradiances were measured using an ILT 1400 Radiometer Photometer with an SEL240 sensor (International Light Technologies, Peabody, MA).

**Figure 7:**
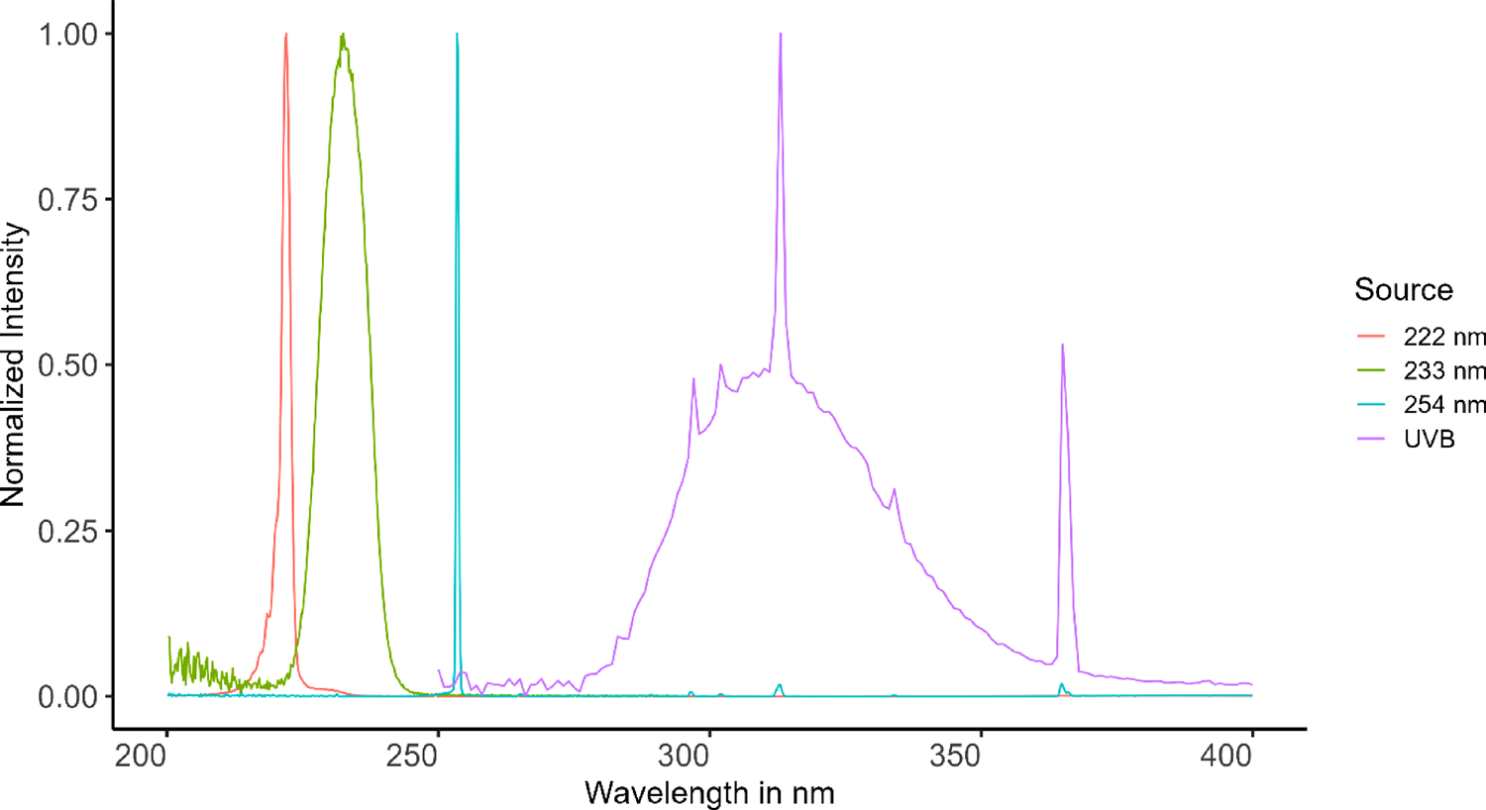
Emission spectra of applied UV light sources. The normalized spectra are shown for 222 nm (red), 233 nm (green), 254 nm (cyan) and broadband UV-B (violet).

Normalized emission spectra of the used UV light sources are shown in Fig. 7.

### Immunohistochemical analysis of DNA damage

Irradiation experiments for analysing DNA damage were performed after shipping the 3D mucosa models in a cooled container from Greifswald to Berlin. In order to semi-quantitatively determine the percentage and location of affected cells, immunostaining was performed on the irradiated tissue samples. The punch biopsies of mucosa models and human oral mucosa were fixated in neutral buffered 4% formalin solution (Sigma #HT501128-4L, Saint Louis, MO) after irradiation. The fixated biopsies were dewatered and embedded in paraffin blocks (Histosec, Merck Millipore)^51^, sectioned to 1–2 µm and incubated with anti-6-4PP (clone 64 M-2, Cosmo Bio), anti-CPD (clone TDM-2, Cosmo Bio) or anti p53 (clone DO7, Novastra) and subsequently stained with Alkaline Phosphatase/RED, Rabbit/Mouse (Agilent Technologies) for the detection of 6-4PP, CPD and p53. Nuclei staining was performed with hematoxylin and slides were cover slipped with Kaiser’s glycerol gelatine (both Merck Millipore). Negative controls were performed by omitting the primary antibody. An AxioImager Z1 microscope (Carl Zeiss MicroImaging, Inc.) was used for histologic documentation in a blinded manner.

### Statistical analysis

Statistical analysis of MTT (*n* = 2–7), TEER (*n* = 2–17) and irritation potential (*n* = 3–4) was done with one-way ANOVA following Dunnett’s multiple comparison test.

DNA damage was measured on human oral mucosa samples of *n* ≥ 3 subjects. Due to low number of cases, a test for normal distribution was omitted and statistical differences between the unrelated groups was performed non-parametrically using a Wilcoxon rank-sum test.

The statistical analysis was executed using R version 4.2.2 (The R Foundation for Statistical Computing, Vienna, Austria) and a significance level of *α* = 0.05 was defined. Display of data was performed in R and OriginPro 2020 (OriginLab Corporation, Northampton, MA). All data are expressed as mean ± standard error (SEM).

## Data Availability

The data is available from the corresponding author Johannes Schleusener (Johannes.schleusener@charite.de) upon reasonable request.
